# Hypoglycemic effects of aqueous persimmon leaf extract in a murine model of diabetes

**DOI:** 10.3892/mmr.2015.3766

**Published:** 2015-05-08

**Authors:** UI-JIN BAE, SOO-HYUN PARK, SU-YOUNG JUNG, BYUNG-HYUN PARK, SOO-WAN CHAE

**Affiliations:** 1 Department of Biochemistry, Chonbuk National University Medical School, Deokjin-gu, Jeonju, Jeonbuk 561–756, Republic of Korea; 2 Clinical Trial Center for Functional Foods, Chonbuk National University Hospital, Deokjin-gu, Jeonju, Jeonbuk 561–712, Republic of Korea; 3 Department of Pharmacology, Chonbuk National University Medical School, Deokjin-gu, Jeonju, Jeonbuk 561–756, Republic of Korea

**Keywords:** water extract of persimmon leaves, α-glucosidase, β-cells, streptozotocin, *db/db*

## Abstract

Previously, powdered persimmon leaves have been reported to have glucose- and lipid-lowering effects in diabetic *(db/db)* mice. As persimmon leaf is commonly consumed as tea, an aqueous extract of persimmon leaves (PLE) was prepared and its anti-diabetic efficacy was investigated. In the present study, PLE was tested for its inhibitory activity on α-glucosidase *in vitro*. An oral maltose tolerance test was performed in diabetic mice. Next, the acute effect of PLE was examined in streptozotocin-induced diabetic mice. Last, the long-term effect of PLE supplementation was assessed in *db/db* after eight weeks. An oral glucose tolerance test, biochemical parameters, as well as histological analyses of liver and pancreas were evaluated at the end of the study. PLE inhibited α-glucosidase activity and increased antioxidant capacity. Streptozotocin-induced diabetic mice pre-treated with PLE displayed hypoglycemic activity. Daily oral supplementation with PLE for eight weeks reduced body weight gain without affecting food intake, enhanced the glucose tolerance during the oral glucose tolerance test (OGTT), improved blood lipid parameters, suppressed fat accumulation in the liver and maintained islet structure in *db/db* mice. Further mechanistic study showed that PLE protected pancreatic islets from glucotoxicity. In conclusion, the results of the present study indicated that PLE exhibits considerable anti-diabetic effects through α-glucosidase inhibition and through the maintenance of functional β-cells. These results provided a rationale for the use of persimmon leaf tea for the maintenance of normal blood glucose levels in diabetic patients.

## Introduction

The prevalence of diabetes in Korean patients has dramatically increased from 1.5 to 9.9% over the last 40 years (1). It is anticipated that the prevalence of diabetes will rise to 11.4% by 2030. This drastic increase in diabetic patients is ultimately associated with secondary micro- and macro-vascular complications (2). Therefore, effective approaches to control blood glucose are required to prevent vascular complications and improve the quality of life of diabetic patients. Initial management often involves lifestyle interventions, including diet and exercise, but in most cases, pharmacotherapy is also required as the disease progresses (2). However, medications used to control blood glucose often cause metabolic side effects, including weight gain and organ toxicity (3,4). Thus, development of alternative therapies is of paramount importance, and natural products that can manage patients’ blood glucose levels without noticeable side effects are gaining considerable attention.

Persimmon *(Diospyros kaki)*, a fruit tree that is native to China and belongs to the family of *Ebenaceae*, is widely distributed in eastern Asia. The fruit of the persimmon tree is consumed as food, whereas the young leaf is mainly used for tea. Persimmon leaf tea contains several bioactive compounds, including flavonoids, triterpenoids, tannins and carotenoids (5–9). A number of studies have reported the beneficial effects of persimmon leaf extract (PLE) on hypertension (5), stroke (10), atherosclerosis (11) and dermatitis (12). Recently, Jung *et al* (13) investigated the metabolic effects of PLE using type 2 diabetic *(db/db)* mice. After oral administration of powdered persimmon leaves for five weeks, glucose- and lipid-lowering effects were observed in the animals, which also led to amelioration of hyperglycemia, dyslipidemia and fatty liver.

In the present study, the anti-diabetic efficacy of PLE in streptozotocin-induced diabetic mice and *db/db* mice was investigated. Furthermore, the underlying mechanism of the anti-diabetic effect of PLE was investigated, particularly focusing on α-glucosidase inhibition and pancreatic β-cell-protecting activities.

## Materials and methods

### Reagents

Unless otherwise stated, all reagents were purchased from Sigma-Aldrich (St. Louis, MO, USA). Collagenase was purchased from Roche Diagnostics (Indianapolis, IN, USA).

### Preparation of PLE

**Figure 1:**
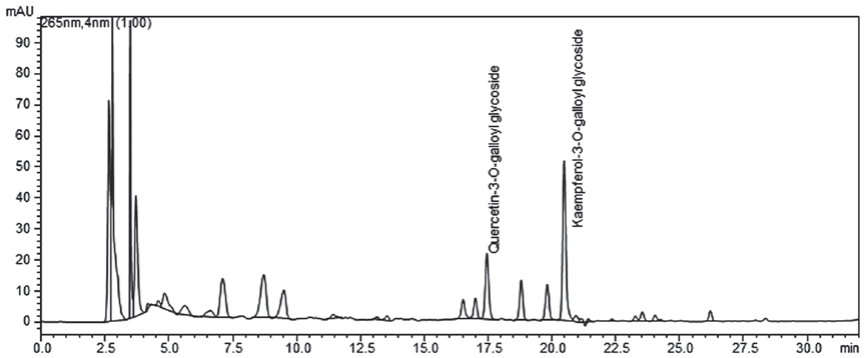
High-performance liquid chromatogram of persimmon leaf extract, which was standardized to contain 7.5 mg quercetin 3-*O*-2′galloylglucoside (and kaempferol 3-*O*-2′galloylglucoside per 1 g extract. Analyses were performed on an X-bridged C18 column with a mobile phase gradient of A) 0.1% formic acid and B) acetonitrile over 50 min. The injection volume was 20 *μ*l. The column temperature was kept constant at 25°C, and the mobile phase flow rate was 1 ml/min with ultraviolet detection at 265 nm.

Persimmon leaves were raised and harvested in Wanju (Jeonbuk, Korea) in June 2013 by Dongsangmyeon Saramdeul Inc. (Jeonbuk, Korea). Persimmon leaves were dried in the shade for one week prior to being powdered and passed through 60-mesh sieves. One volume of persimmon leaf powder was added to 10 volumes of distilled water and extracted at 90–100°C for 3 h. The aqueous phase was filtered and concentrated with a vacuum evaporator (Eyela, Japan). After lyophilization, the powder was stored at −80°C until used. The components of PLE were analyzed by the Development Institute of Traditional Korean Medicine (Jeonnam, Korea) using a high-pressure liquid chromatography workstation (Shimadzu, Japan) (Fig. 1). Analyses were performed on an X-bridged C18 column with a mobile phase gradient of A) 0.1% formic acid and B) acetonitrile over 50 min. Gradient elution was programmed at a flow rate of 0.25 ml/min as follows: 0 min (100%), 10 min (90%), 30 min (40%), 45 min (30%) and 50 min (90%). The injection volume was 20 *μ*l. The column temperature was kept constant at 25°C, and the mobile phase flow rate was 1 ml/min with ultraviolet detection at 265 nm. PLE was standardized to contain 4–7 mg total quercetin 3-*O*-2′galloylglucoside (C_24_H_24_O_19_) and kaempferol 3-*O*-2′galloylglucoside (C_24_H_24_O_18_) per 1 g of extract.

### In vitro α-glucosidase assay

Yeast α-glucosidase (0.5 U) dissolved in 0.2 M potassium phosphate buffer (pH 6.8) was mixed with various concentrations of PLE or acarbose. After incubation at 37°C for 15 min, 3 mM *p*-nitrophenyl-α-D-gluc opyranoside was added. The reaction was further incubated at 37°C for 10 min and then stopped by the addition of 0.1 M Na_2_CO_3_. The absorption (Abs) of 4-nitrophenol was measured at 405 nm. Reaction mixture without any sample was used as a control, and the mixture without substrate was used as a blank. The percent inhibition of α-glucosidase was calculated as [1-(Abs_sample_−Abs_blank_)/Abs_control_]×100. Measurements were performed in triplicate.

### Oxygen free radical scavenging assay

The anti-oxidant activity of each sample extract was assessed by the ability of the extract to scavenge 2,2-diphenyl-1-picrylhydrazyl (DPPH) free radicals. The extracts, in separate test tubes, were allowed to react with DPPH. DPPH free radical scavenging activity was monitored by measuring the decline in absorbance at 517 nm. Butylated hydroxyanisole was used as the standard compound.

### Experimental design

Pathogen-free, male C57BL/6 mice were purchased from Orientbio (Sungnam, Korea). The mice were housed at 20°C with 50% relative humidity, a 12-h light/dark cycle (light from 6:00 am to 6:00 pm) and were provided free access to drinking water. To induce diabetes, eight-week-old male C57BL/6 mice were injected via the tail vein with 100 mg/kg body weight streptozotocin (STZ) dissolved in 0.1 mol/l sodium citrate buffer (pH 4.0). The control mice received citrate buffer alone. PLE (50 or 250 mg/kg body weight) was injected daily for five days via oral gavage prior to administration of STZ. STZ was first administered on day one. On day six, mice were sacrificed by decapitation without anesthesia and trunk blood was collected.

In addition, seven-week-old male C57BL/KsJ-*db/db* (*db/db*) mice were purchased from the Jackson Lab (Bar Harbor, ME, USA) and fed a normal chow diet. Starting at eight weeks of age, the point at which the mice become diabetic, the *db/db* mice were treated with PLE (50 or 250 mg/kg) for eight weeks via oral gavage once daily. Each group was made up of five mice. As a positive control, acarbose (10 mg/kg) was administered instead of PLE. Food consumption and body weight were recorded every week. At the end of the experimental period, an oral glucose tolerance test (OGTT; 1 g/kg body weight) was performed. After a 14 h fast, glucose was administered by oral gavage (2 mg/g). The blood glucose level was subsequently determined from the tail vein at 0, 15, 30, 60 and 120 min following the glucose administration. Animals were sacrificed by decapitation, after which blood samples were collected, and livers were removed and weighed. All of the animal experiments were performed in accordance with the Guide for the Care and Use of Laboratory Animals published by the US National Institutes of Health (NIH Publication no. 85-23, revised 2011). The protocol of the present study was approved by the Institutional Animal Care and Use Committee of Chonbuk National University (permit no. CBU 2014-00048).

### Oral maltose tolerance test in streptozotocin-induced diabetic mice

Mice were classified into four groups (1–4) containing five mice each. Groups 1 and 2 received phosphate-buffered saline (PBS) as a negative control or acarbose (3 mg/kg) as a positive control, respectively. Groups 3 and 4 were treated with PLE at two doses (50 and 250 mg/kg). All samples were administered orally to 12-h fasted mice, and 3 g/kg of maltose was administered 5 min thereafter. Blood was collected from the tail vein at 0, 15, 30, 60 and 120 min after loading maltose.

### Biochemical analyses

Blood glucose levels were measured by Accu-Chek Aviva glucose monitors (Roche Diagnostics, Indianapolis, IN, USA) and plasma insulin was measured using an ELISA kit (cat. no. EZRMI-13K; Millipore, Bedford, MA, USA). Plasma levels of total cholesterol (TC), triglyceride (TG) and HDL-cholesterol were measured using commercially available kits (cat. no’s. AM202-K, AM157S-K and AM203-K, respectively; Asan Pharmaceutical, Seoul, Korea). For liver TG quantification, liver tissues were homogenized and extracted in chloroform, methanol and DW (2/1/1 ratio).

### Histology

Tissues were removed and immediately placed in 10% formalin solution, embedded in paraffin and cut into 5-*μ*m sections. Specimens were stained with hematoxylin and eosin (H&E) to identify morphological changes. For immunohistochemical analysis, tissue sections were subjected to a microwave antigen retrieval procedure (1,000 watts for 5 min; CPC-600; Cuisinart, East Windsor, NJ, USA) in 0.01 mol/l sodium citrate buffer. After blocking endogenous peroxidase, the sections were incubated with Protein Block Serum-Free (DAKO, Glostrup, Denmark) to block non-specific staining and then with rabbit anti-insulin antibody (cat. no. sc-9168; 1:100; Santa Cruz Biotechnology, Dallas, TX, USA) for 12 h at 4°C. Peroxidase activity was detected using 3-amino-9-ethyl carbazole. Tissue sections were observed using a light microscope (Eclipse E600 polarizing microscope; Nikon, Tokyo, Japan).

### Islet isolation and glucose-stimulated insulin secretion (GSIS) assay

Pancreatic islets were isolated from 12-week-old mice using the collagenase digestion method as previously described (14). Following isolation, islets were cultured overnight in RPMI-1640 supplemented with 2 mM L-glutamine, 10% heat-inactivated fetal calf serum, 100 units/ml penicillin and 100 *μ*g/ml streptomycin in humidified air containing 5% CO_2_ at 37°C. Prior to experiments, islets were washed three times in RPMI-1640 and cultured overnight. After the initial culture period, islets were cultured for three days in identical RPMI-1640 containing 5.5 or 30 mmol/l glucose and subsequently washed three times in Krebs-Ringer bicarbonate buffer [25 mM 4-(2-hydroxyethyl)-1-piperazineethanesulfonic acid, 115 mmol/l NaCl, 24 mmol/l NaHCO_3_, 5 mmol/l KCl, 1 mmol/l MgCl_2_, 2.5 mmol/l CaCl_2_ and 0.1% bovine serum albumin, pH 7.4] containing 2.8 mmol/l D-glucose. Insulin secretion assays were performed with 2.8 or 16.7 mmol/l glucose and measured using an ELISA kit (cat. no. EZRMI-13K; Millipore, Bedford, MA, USA).

### Statistical analysis

Statistical analysis was performed using analysis of variance and Duncan’s tests on through GraphPad Prism v5.02 (GraphPad Software Inc., La Jolla, CA, USA). P<0.05 was considered to indicate a statistically significant difference between values.

## Results

### PLE inhibits α-glucosidase activity

**Figure 2:**
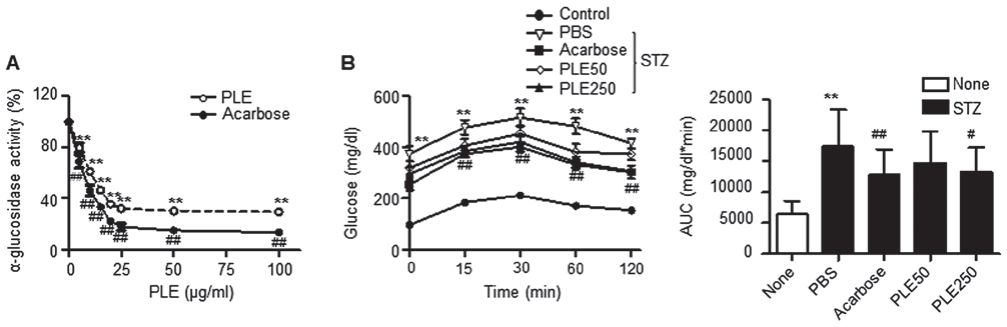
α-Glucosidase inhibitory activity of PLE. (A) Effects of different concentrations of PLE on α-glucosidase activity *in vitro* (^**^P<0.01, ^##^P<0.01, vs. no treatment). (B) Effects of PLE on blood glucose levels after administration of 3 g/kg maltose in STZ-induced diabetic mice. Values are expressed as the mean ± standard error of the mean (n=5; ^**^P<0.01, vs. control mice; ^#^P<0.05, ^##^P<0.01, vs. PBS-treated STZ mice). PLE50, persimmon leaf extract (50 mg/kg); PLE250, persimmon leaf extract (250 mg/kg); STZ, streptozotocin; PBS, phosphate-buffered saline; AUC, area under curve.

The *in vitro* inhibitory activity of PLE against yeast α-glucosidase is shown in Fig. 2A. PLE inhibited α-glucosidase activity in a dose-dependent manner and therefore should be considered an effective α-glucosidase inhibitor: PLE at a concentration of 100 *μ*g/ml inhibited α-glucosidase activity by 70.5% and was 16.0% less potent than acarbose, which was used as a positive control. The half maximal inhibitory concentration (IC_50_) value on α-glucosidase activity was 4.7 *μ*g/ml.

The results of the maltose tolerance experiment are presented in Fig. 2B. After the administration of maltose, the blood glucose levels in normal mice increased after 15 min. This elevation was statistically significant compared with the levels at time 0 for each group. At 120 min, the blood glucose levels returned to their basal values. Oral administration of 250 mg/kg PLE inhibited the increases in glucose levels after 30 min, which was statistically significant compared with levels in the corresponding controls at each time-point. The administration of acarbose also significantly decreased the increase of postprandial blood glucose. The areas under the curve (AUC) for glucose were reduced by 15.34% by PLE at 50 mg/kg, 23.63% by PLE at 250 mg/kg and 26.51% by acarbose.

**Figure 3:**
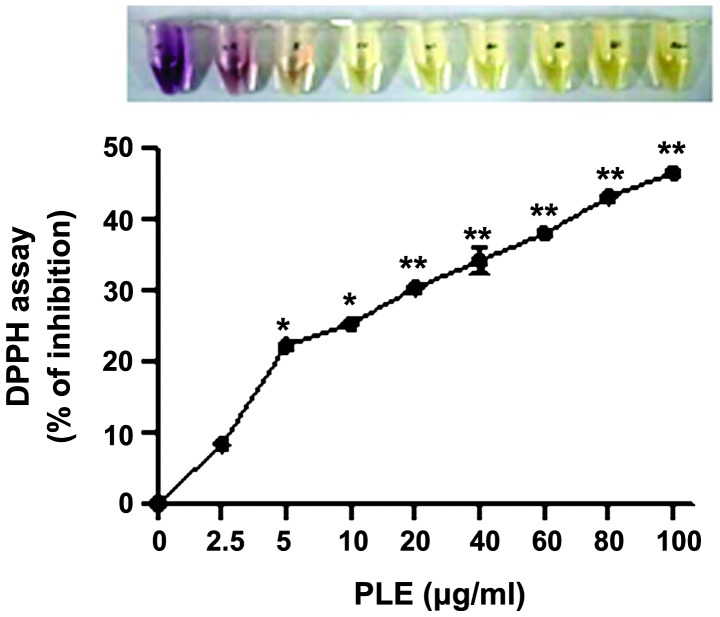
Free-radical scavenging activity of PLE using the cell-free DPPH assay. Values are expressed as the mean ± standard error of the mean of three independent experiments (^*^P<0.05, ^**^P<0.01, vs. no treatment). PLE, persimmon leaf extract; DPPH, 2,2-diphenyl-1-picrylhydrazyl radical.

Furthermore, the anti-oxidant properties of PLE were determined using the cell-free DPPH assay. PLE inhibited DPPH activity in a dose-dependent manner (Fig. 3), which confirms the findings of a previous study (15).

### PLE protects mice against STZ-induced diabetes

**Figure 4:**
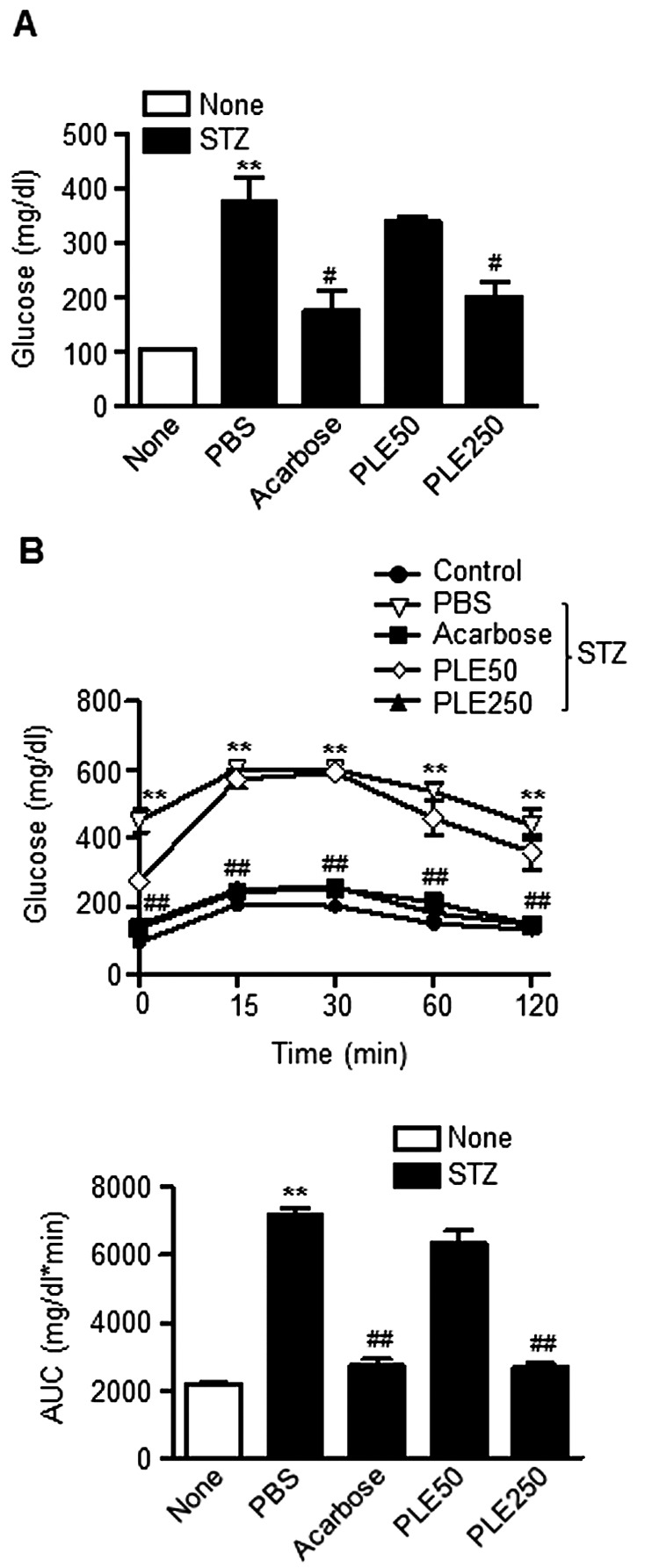
Improved glycemic control in STZ-induced hyperglycemic mice after short-term treatment with PLE. PLE50 or PLE250 was administered via oral gavage daily for five days prior to the administration of STZ. (A) Six days following STZ injection, levels of post-prandial glucose were determined. (B) Oral glucose tolerance tests – five days after STZ injection, glucose (1 g/kg) was administered to overnight-fasted mice and plasma glucose concentrations were measured. The bar graph represents areas under the curve. Values are expressed as the mean ± standard error of the mean (n=5). ^**^P<0.01 vs. control mice; ^#^P<0.05, ^##^P<0.01 vs. PBS-treated STZ mice. PLE50, persimmon leaf extract (50 mg/kg); PLE250, persimmon leaf extract (250 mg/kg); STZ, streptozotocin; PBS, phosphate-buffered saline; AUC, area under curve.

Powdered persimmon leaf has previously been reported to have glucose-and lipid-lowering effects in *db/db* mice (13). First, the present study evaluated the hypoglycemic effects of PLE at concentrations of 50 mg/kg (low-dose) and 250 mg/kg (high-dose) in STZ-induced diabetic mice. As shown in Fig. 4A, a single intravenous injection of STZ induced the increase of fasting blood glucose levels to ~400 mg/dl. However, the fasting glucose levels were significantly reduced to 176 mg/dl in the STZ mice pre-treated with high-dose PLE. The group of mice treated with acarbose (10 mg/kg) showed similar blood glucose levels. Of note, the body weight among the groups did not change over the course of the study (data not shown).

The effect of PLE on the post-prandial increase in blood glucose in STZ-induced diabetic mice was determined via OGTT Consistent with the above results, oral administration of high-dose PLE significantly prevented an increase in plasma glucose levels (Fig. 4B). High-dose PLE and acarbose decreased the AUCs for the postprandial glucose responses by 61.6 and 62.4%, respectively, compared with that in the PBS-treated group (P<0.01). These results indicated that treatment with PLE prevents STZ-induced β-cell damage in mice.

### Long-term treatment with PLE ameliorates hyperglycemia and dyslipidemia in db/db mice

**Figure 5:**
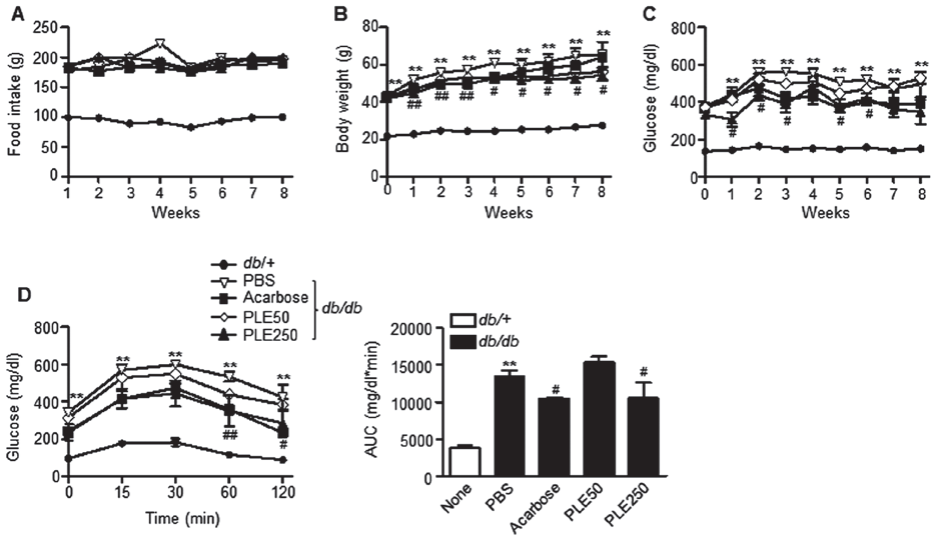
Improved glycemic control in *db/db* mice after eight weeks of treatment with PLE. Mice received daily oral supplementation with PLE50 or PLE250 or 10 mg/kg acarbose for eight weeks. (A) Food intake and (B) body weight change were recorded at indicated times. (C) Post-prandial blood glucose levels. (D) Plasma glucose concentrations during oral glucose tolerance tests in overnight-fasted mice. The bar graph represents areas under the curve. Values are expressed as the mean ± standard error of the mean (n=5). ^**^P<0.01 vs. control mice; ^#^P<0.05, ^##^P<0.01 vs. PBS-treated *db/db* mice. PLE50, persimmon leaf extract (50 mg/kg); PLE250, persimmon leaf extract (250 mg/kg); PBS, phosphate-buffered saline; *db/db*, diabetic; AUC, area under curve.

To further evaluate the therapeutic effects of PLE on diabetes, the long-term anti-diabetic effects of PLE in *db/db* mice were evaluated. Food intake was not significantly influenced by PLE treatment (Fig. 5A); however, the body weight was significantly decreased by high-dose PLE treatment (Fig. 5B). Diabetic *db/db* mice at 16 weeks of age that were fed normally had hyperglycemia with postprandial blood glucose levels of ~494 mg/dl (Fig. 5C). High-dose PLE decreased postprandial blood glucose levels in *db/db* mice as effectively as acarbose. The decrease in blood glucose levels by PLE appeared to be dose-dependent, as low doses of PLE (50 mg/kg) revealed no glucose lowering effects, whereas high doses of PLE (250 mg/kg) significantly decreases glucose levels (P< 0.01).

An OGTT was performed to determine the effects of PLE on glucose tolerance after eight weeks of PLE treatment (Fig. 5D). The AUC for glucose response of high-dose PLE-treated *db/db* mice was significantly lower than that of the (*db/db*) mice. By contrast, oral administration of low-dose PLE did not show any improvement in glucose tolerance.

**Figure 6:**
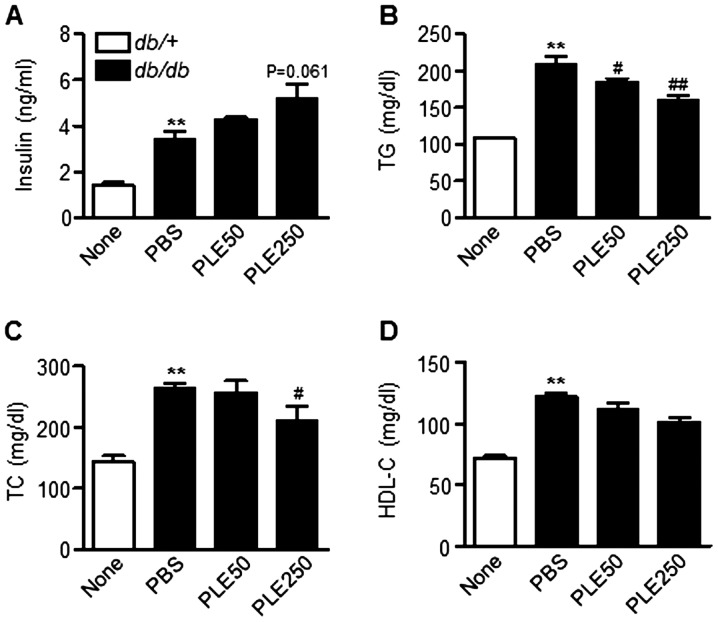
Biochemical parameters in *db/db* mice after eight weeks of treatment with persimmon leaf extract. Mice received daily oral supplementation with PLE50 or PLE250. At the end of the study, plasma concentrations of (A) insulin, (B) TG, (C) TC and (D) HDL-C were determined. Values are expressed as the mean ± standard error of the mean (n=5). ^**^P<0.01 vs. control mice; ^#^P<0.05, ^##^P<0.01 vs. PBS-treated *db/db* mice. TG, triglyceride; TC, total cholesterol; HDL-C, high-density lipoprotein cholesterol; PLE50, persimmon leaf extract (50 mg/kg); PLE250, persimmon leaf extract (250 mg/kg); PBS, phosphate-buffered saline; *db/db*, diabetic.

Plasma insulin levels between *db/db* mice that received PBS and those that had been administered PLE were also compared. The serum insulin levels in high-dose PLE-treated mice tended to be higher than those in PBS-treated mice (Fig. 6A). In addition, as shown in Fig. 6B and C, high-dose PLE significantly lowered plasma TG (160.5±15.9 mg/dl; P<0.01) and TC levels (209.2±74.5 mg/dl; P<0.05) compared with those in the control mice (208.9±32.4 and 263.9±19.3 mg/dl, respectively). Plasma HDL-cholesterol levels were not significantly different among groups (Fig. 6D). Overall, these results suggested that PLE has glucose- and lipid-lowering effects in *db/db* mice.

### Long-term treatment with PLE prevents fatty liver development in db/db mice

**Figure 7:**
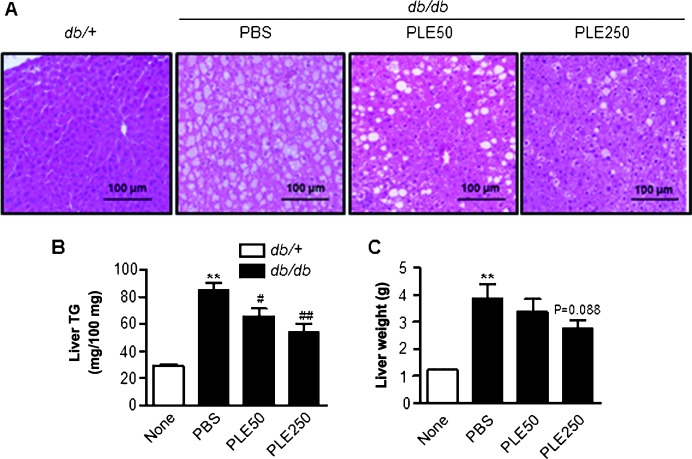
Prevention of liver steatosis in *db/db* mice after eight weeks of treatment with PLE. (A) Representative hematoxylin and eosin-stained histological sections of liver from mice (magnification, ×200). (B and C) Liver TG and wet weight were determined. Values are expressed as the mean ± standard error of the mean (n=5). ^**^P<0.01 vs. control mice; ^#^P<0.05, ^##^P<0.01 vs. PBS-treated *db/db* mice. PLE50, persimmon leaf extract (50 mg/kg); PLE250, persimmon leaf extract (250 mg/kg); PBS, phosphate-buffered saline; *db/db*, diabetic; TG, triglycerides.

As diabetes can trigger hepatic steatosis, livers were assessed for the extent of fat accumulation. The results indicated that PLE supplementation was associated with less steatotic livers (data not shown). Examination of H&E-stained sections demonstrated marked macrovesicular steatosis in *db*/*db* mice, and the degree of hepatic steatosis was markedly alleviated by PLE (Fig. 7A). Liver TG and liver weight were concordant with the histological findings (Fig. 7B and C). Treatment of *db/db* mice with high-dose PLE resulted in significant decreases in hepatic TG contents (85.3±12.5 mg/100 mg vs. 54.2±15.8 mg/100 mg; P<0.01) and liver weight (3.8±0.8 vs. 2.7±0.6 g; P=0.088) as compared with that in the control *db/db* mice.

### Long-term treatment with PLE protects pancreatic β-cells in db/db mice

**Figure 8:**
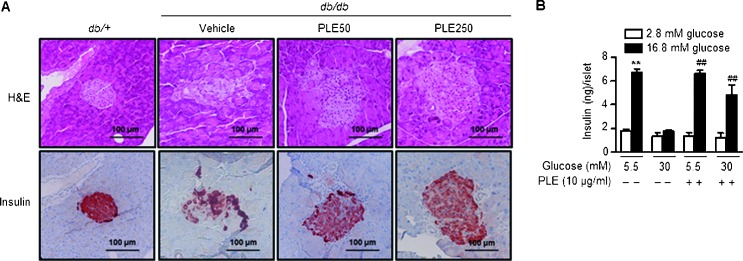
Prevention of pancreatic islet destruction in *db/db* mice after eight weeks of treatment with PLE. (A) Sections of the pancreata of the mice were stained with H&E or immunostained with insulin antibody (magnification, ×200). (B) Mouse islets were treated with 5.5 or 30 mM glucose with or without PLE. Following a three-day incubation, insulin secretion assays were performed with 2.8 or 16.7 mmol/l D-glucose. Values are expressed as the mean ± standard error of the mean (n=5). ^**^P<0.01 vs. control islets; ^#^P<0.05, ^##^P<0.01 vs. 30 mM glucose-incubated islets. H&E, hematoxylin and eosin; PLE50, persimmon leaf extract (50 mg/kg); PLE250, persimmon leaf extract (250 mg/kg); PBS, phosphate-buffered saline; *db/db*, diabetic.

As PLE administration increased plasma insulin levels (Fig. 6A), pancreata were examined using H&E staining. Pancreatic islets of diabetic *db/db* mice exhibited degeneration and poorly defined margins (Fig. 8A). Immunostaining with an insulin antibody showed weak insulin staining. By contrast, pancreatic islets of high-dose PLE-treated mice had a round shape and high insulin immunoreactivity, suggesting the protection of β-cells by PLE.

To determine the protective effects of PLE on β-cells, the present study examined whether it was effective in protecting pancreatic islets from glucotoxicity. Islets from mice were isolated and incubated under normal glucose (5.5 mM) or high-glucose (30 mM) culture conditions. Basal and glucose-stimulated insulin secretion was assessed after three days of culture (Fig. 8B). Results showed that the amount of glucose-stimulated insulin secretion was 6.65±0.48 ng/islet/h in normal glucose-cultured islets and 4.80±1.48 ng/islet/h in high-glucose-cultured islets. Following pre-treatment with PLE, however, the insulin secretion under high-glucose conditions was significantly restored to a level closer to the control value. Basal insulin release among the groups was similar. In conclusion, *in vitro* and *in vivo* results of the present study suggested that PLE exhibits protective effects on β-cells.

## Discussion

In the present study, PLE was shown to improve the biochemical parameters of glucose and lipid metabolism and prevented fatty liver development in *db/db* mice after eight weeks of oral supplementation. Similar results have been reported by a study of five-week treatment with powdered persimmon leaves (13), suggesting that long-term oral supplementation with persimmon leaf can effectively exert glycemic control in diabetic mice. In addition, five-day oral supplementation with PLE prevented diabetes development in STZ-treated mice. In recent meta-analysis studies, flavonoids have been reported to have acute and chronic effects on glucose and lipid metabolism (16,17). As PLE contains a considerable amount of flavonoids, including quercetin and kaempferol (4), it is reasonable to expect PLE to exhibit acute as well as chronic anti-diabetic effects. However, even though PLE possesses hypoglycemic effects, the blood glucose levels in PLE-supplemented *db/db* mice were still higher throughout the experimental period than those of wild-type db/m mice, suggesting that PLE causes a certain improvement in glucose tolerance under hyperglycemic conditions.

Although the body weight decreased in *db/db* mice after PLE supplementation, a similar decrease in food intake was not observed. This finding is in contrast with results from a study by Jung *et al* (13), in which oral administration of the powder of persimmon leaves caused significant decreases in body weight gain and food intake compared to those in control group mice. This discrepancy may result from a difference in persimmon leaf sources between the two studies. Furthermore, the present study used PLE, whereas Jung *et al* fed mice with 5% powder of persimmon leaves. As the aqueous extract and powdered persimmon leaves display differences in their chemical composition, their application is likely to have different outcomes. In the present study, the conclusion that PLE reduced body weight and/or improved glucose tolerance simply by reducing food intake can be excluded.

Of note, the hypoglycemic effect of PLE in *db/db* mice was observable as early as one week after PLE supplementation. This finding led to the hypothesis that α-glucosidase and α-amylase inhibition are possible underlying mechanisms of the anti-diabetic effects of PLE, as these enzymes are involved in the digestion of complex carbohydrates from food into absorbable monosaccharides (18). Accordingly, an *in vitro* study was performed to examine the effects of PLE on α -glucosidase activity. The results revealed that PLE inhibited -glucosidase activity in a dose-dependent manner with an IC_50_ value of 4.7 *μ*g/ml. In the following oral maltose tolerance test in normal mice, PLE showed marked α-glucosidase inhibitory activity. In addition, a study by Kawakami *et al* (9) reported α-amylase inhibitory activity of persimmon leaves. Therefore, inhibition of α-glucosidase and/or α-amylase by PLE may prolong overall digestion time, causing a delay in glucose absorption, consequently reducing the rapid increase of postprandial blood glucose.

PLE significantly suppressed the increase in the post-prandial blood glucose levels as compared to those in PBS-treated *db/db* mice. Insulin has a pivotal role in maintaining the post-prandial glucose levels within a normal range by enhancing glycogen synthesis and glycolysis, and by suppressing gluconeogenesis (19). In general, the *db/db* mice exhibited an initial phase of hyperinsulinemia to compensate for insulin resistance and progressively develop insulinopenia with age, a characteristic commonly observed in patients during late stages of type 2 diabetes (20). In *db/db* mice supplemented with PLE, the plasma insulin levels were higher than those in PBS-treated *db/db* mice. In addition, islet architecture was relatively well preserved and the mass of insulin-immunoreactive β-cells was increased in PLE-supplemented mice, suggesting that PLE-supplemented *db/db* mice still displayed insulin-secreting β-cell masses. This assumption was evidenced by *ex vivo* experiments using isolated islets. Assessment of insulin secretion capacities after culturing of islets under glucotoxic conditions showed that glucose-stimulated insulin secretion was increased in PLE-treated islets as compared with that in untreated islets.

In conclusion, the present study provided further evidence for the anti-diabetic efficacy of PLE in STZ-induced diabetic mice and *db/db* mice, which was comparable to the effect elicited by acarbose. Glucose tolerance during OGTT was enhanced, lipid parameters were improved and fat accumulation in the liver was suppressed in PLE-supplemented mice compared to those of the control mice. These beneficial effects are at least partially mediated via suppression of α-glucosidase activity and preserved, functional β-cell masses. The former leads to decreased blood glucose levels and the latter leads to increased insulin levels. Therefore, the results of the present study implied that supplementing pre-diabetes or diabetes patients with PLE may be a way to maintain blood glucose levels within a normal range.
